# Cadmium and Lead Concentration in Drinking Instant Coffee, Instant Coffee Drinks and Coffee Substitutes: Safety and Health Risk Assessment

**DOI:** 10.1007/s12011-022-03129-2

**Published:** 2022-01-25

**Authors:** Anna Winiarska-Mieczan, Karolina Jachimowicz, Svitlana Kislova, Małgorzata Kwiecień, Zvenyslava Zasadna, Dmytro Yanovych

**Affiliations:** 1 Institute of Animal Nutrition and Bromatology, University of Life Sciences in Lublin, Akademicka 13, 20-950 Lublin, Poland; 2 State Scientific-Research Control Institute of Veterinary Medical Products and Fodder Additives, Lviv, Ukraine

**Keywords:** Instant coffee, Coffee substitutes, Cadmium, Lead, Safety

## Abstract

The presence of heavy metals in food is a global problem. The paper aimed to examine the content of cadmium (Cd) and lead (Pb) in instant coffee and instant coffee substitutes. The safety of consumption of the beverages by adult Poles was estimated based on the following parameters: tolerable weekly intake (TWI) %, benchmark dose lower confidence limit (BMDL) %, chronic daily intake (CDI), target hazard quotient (THQ) and hazard index (HI), for three beverage consumption patterns—one, two or three servings a day. Forty-nine samples of coffee, instant coffee drinks and coffee substitutes were analysed. The content of cadmium and lead was determined by ICP (inductively coupled plasma) analysis. The maximum level of Cd in the analysed beverages was 3.2 µg, and that of Pb was 82.6 µg per 1 kg. The tolerable level of intake of Cd (TWI) and Pb (BMDL) with the analysed beverages did not exceed 2.5%. The value of CDI, THQ and HI was not higher than 1, which means that the risk of diseases related to chronic exposure to Cd and Pb consumed with coffee should be evaluated as very low. However, special note should be taken of Pb, as the level of this metal was higher than that of Cd, and for beverages with a higher weight per serving (e.g. Cappuccino), the intake of Pb can exceed consumer-safe levels if they are consumed on a regular basis. Therefore, it should be considered whether it is advisable for flavoured multi-ingredient instant coffee drinks to be consumed from time to time only, and natural coffee with optional milk and/or sugar be the choice of regular coffee drinkers.

## Introduction

Coffee is one of the most popular beverages around the world. Its popularity is due to both its flavour and aroma [1] and proven health benefits related to the presence of active antioxidants such as polyphenols, caffeine and diterpenes [2]. However, it is important to note the association of diterpenes with elevated plasma cholesterol and triglyceride levels, mainly through an increase in plasma low-density lipoprotein (LDL) [2]. Regular drinking of moderate amounts of coffee (not more than 5 cups a day) protects the body against DNA damage and mitigates the risk of developing breast, prostate and colorectal cancer and many other chronic diseases (e.g. type 2 diabetes, Parkinson’s, depression) [3–7]. More than 95% of adults in Poland, 61% in Italy and 78% in Spain are regular coffee drinkers [1, 6, 8]. Statistically, in 2019 in Poland, one person consumed 2.16 kg of coffee, that is, 0.18 kg per month [9]. This level of consumption has remained steady for several years. Poles most often drink 1–3 cups of coffee a day [1], Columbians on average 3.4 cups a day [10] and Ethiopians 4 cups a day [11]. Our surveys showed that in Poland, instant coffee was the choice of 32% of respondents, whereas 23% declared drinking beverages such as instant coffee drinks of the ‘2-in-1’ or ‘3-in-1’ type, and decaffeinated coffee, cereal coffee and cappuccino [1]. Similarly, Chudy [8] and Czarniecka-Skubina et al. [12] demonstrated that about 50% of coffee consumers in Poland drink instant coffee and coffee blends. Coffee beverages and flavoured coffees are most often the choice of young people aged 18–25 [12]. Instant coffee owes its popularity primarily to the fact that it is easy and quick to prepare. For many years, the trend of choosing instant coffee has remained high in Poland, despite the fact that natural coffee is perceived as real coffee by respondents, while instant coffee and coffee blends are regarded as non-natural and high-calorie products [13]. The difference between ground coffee and instant coffee is the content of caffeine. When one teaspoon of ground coffee is brewed, the cup contains 40 mg of caffeine, and for one teaspoon of instant coffee, 60 mg [14]. Moreover, instant coffee contains more polyphenols—ca. 61 mg per 1 g, and ground coffee less than 19 mg per 1 g only. This difference is due to the processing method. Instant coffee has a higher content of minerals (Ca, Mg, Zn, Fe, Mn, Al, Cr and Ni) compared to the same amount of ground coffee [15].

Unfortunately, coffee also contains toxic heavy metals such as cadmium (Cd) and lead (Pb) [16–18], although available studies primarily cover instant coffee. The presence of heavy metals in food is a global problem. Despite available studies showing that the levels of Cd and Pb in food are normally safe for humans, heavy metals accumulate in the tissues of living organisms and have a long half-life (5–30 years for Cd and 30 days in soft tissue and up to 10 years in bones for Pb) [19], so they pose a risk to health when supplied regularly, even in small amounts. Both Cd and Pb display a strong teratogenic, carcinogenic, mutagenic and embryotoxic effect [20]. As recommended by EFS, the tolerable weekly intake (TWI) of Cd should not exceed 2.5 μg kg^−1^ of body weight per week [21], and the benchmark dose lower confidence limit (BMDL) of Pb should not exceed 10.5 μg kg^−1^ of body weight per week (BMDL01—atherogenic effect of Pb) and 4.4 μg kg^−1^ of body weight per week (BMDL10—nephrotoxic effect of Pb) [22]. The content of heavy metals is usually analysed in ground coffee; however, since the consumption of instant coffee and instant coffee drinks and that of natural coffee is comparatively high in Poland [1, 8, 12], it is essential to evaluate whether it is safe to drink such beverages. The study aimed to measure the content of cadmium and lead in instant coffee, coffee drinks and instant coffee substitutes. In addition, it was also evaluated whether coffee drinks are safe for adult Poles in terms of their Cd and Pb content. The presented results form part of a project estimating the intake of minerals (toxic and essential) by the population of Poland.

## Material and Methods

### Study Material

Forty-nine samples of coffee, instant coffee drinks and coffee substitutes were analysed (Table 1). The coffee was bought at grocery stores in Chełm, Zamość and Lublin (eastern Poland) in August 2019. The samples were stored in sealed original packaging at room temperature until analyses.

**Table 1:** Quality control

	Cd	Pb
Blank sample	1 M HNO_3_	1 M HNO_3_
Certified reference material (1)	INCT-TL-1 (Tea leaves)	INCT-TL-1 (Tea leaves)
Certified reference material (2)	INCT-MPH-2 (Mixed Polish herbs)	INCT-MPH-2 (Mixed Polish herbs)
Certified element concentration in CRM 1		
Certified, mg kg^−1^	0.030	1.78
Observed, mg kg^−1^	0.029	1.76
Recovery rate, %	98	99
Certified element concentration in CRM 2		
Certified, mg kg^−1^	0.199	2.16
Observed, mg kg^−1^	0.189	2.22
Recovery rate, %	95	103
Precision, %	6.04	6.07
Replicates	3	3

### Chemical Analysis

#### Preparation of Samples for Analysis

The samples were averaged by manual mixing. A sample of about 3 g was weighed in three replications into previously heat-sterilised china crucibles and then subjected to dry mineralisation in a muffle furnace at a temperature of 550 °C using hydrogen peroxide as an antioxidant—as described elsewhere [20]. The resulting ash was dissolved in 10 mL of 1 M HNO_3_.

#### Determination of the Content of Cd and Pb

The content of cadmium and lead was determined by ICP (inductively coupled plasma mass spectrometry) in a Varian 820 MS Mass Spectrometer (Varian, Melbourne, Australia). Determination conditions were as follows: mass monitored 114 (Cd), 206, 207, 208 (Pb); plasma, argon; plasma gas flow 1.7 L min^−1^; RF power 1.37 kW. The calibration curve was drawn using the following models: Cd (99.99% purity; concentration of solutions 0.2, 0.4, 1, 2, 4 and 10 µg Cd L^−1^ 1% HNO_3_), and Pb (99.99% purity; concentration of solutions 0.1, 0.2, 0.5, 1, 2 and 5 µg Pb L^−1^ 1% HNO_3_). The results of measurements were verified against a blank sample (1 M HNO_3_) and certified reference materials—INCT-TL-1 Tea leaves (0.030 mg Cd and 1.78 mg Pb per 1 kg) and INCT-MPH-2 Mixed Polish herbs (0.199 mg Cd and 2.16 mg Pb per 1 kg). The percentage of Cd and Pb recovered from the reference materials was 95–103%, with the measurement precision being 6.04 for Cd and 6.07 for Pb (Table 1). The limit of detection (LOD) was 0.004 µg kg^−1^ for Cd and 0.005 µg kg^−1^ for Pb. The limit of quantification (LOQ) was 0.01 µg kg^−1^ for Cd and 0.030 µg kg^−1^ for Pb. Each chemical analysis was repeated three times.

#### Reagents and Reference Materials

Nitric acid (65% ultra-pure HNO_3_) and hydrogen peroxide (30% pure H_2_O_2_) were purchased from POCH S.A. (Poland). The Cd and Pb standards were purchased from Merck (Germany). The certified reference materials INCT-TL-1 and INCT-MPH-2 were purchased from the Institute of Nuclear Chemistry and Technology (Warsaw, Poland).

#### Calculations

The safety of drinking beverages was calculated for three patterns of consumption: one serving, two servings or three servings a day for 365 days in a year [1, 18]. One serving was described as the quantity of the instant product recommended by manufacturers, as indicated on the packaging. For beverages in bulk packaging, one teaspoon of the powder was weighed, and for sachets, one serving corresponded to one sachet (Table 2).

**Table 2:** Characteristic of the analysed instant coffee

Type of coffee	Coffee varieties	Trade mark	Size of package, g	Size of portion, g	Content of coffee	Made in
Instant coffee	No data	A-1	200	2.8	Instant coffee 100%	Holland
Instant coffee	Arabica + Robusta	B-1	200	3.0	Instant coffee 100%	Poland
Instant coffee	No data	B-2	200	2.9	Instant coffee 100%	Poland
Instant coffee	Arabica	C-1	100	3.1	Instant coffee 100%	Poland
Instant coffee	No data	D	500	2.9	Instant coffee 100%	Poland
Instant coffee	Arabica	E-1	100	3.0	Instant coffee 100%	Germany
Instant coffee	Robusta	E-2	200	2.6	Instant coffee 100%	Germany
Instant coffee	No data	F	50	3.0	Instant coffee 100%	Germany
Instant coffee	No data	C-2	2.0	2.0	Instant coffee 100%	Poland
Instant coffee	Arabica	G	1.8	1.8	Instant coffee 100%	Poland
Instant coffee	Arabica + Robusta	C-3	2.0	2.0	Instant coffee 100%	Poland
Blend of green and roasted instant coffee	No data	B-3	20	3.2	Roasted instant coffee (65%), green coffee (35%)	Poland
Blend of green and roasted instant coffee	No data	B-4	100	3.1	Roasted instant coffee (65%), green coffee (35%)	Poland
Blend of green and roasted instant coffee	No data	C-4	12	12.0	Roasted instant coffee (12%), green coffee (4%)	Poland
Blend of green and roasted instant coffee	No data	H	500	3.3	Roasted instant coffee (65%), green coffee (35%)	Poland
Blend of green and roasted instant coffee	Robusta	C-5	20	20.0	Roasted instant coffee (65%), green coffee (35%)	Poland
Blend of green and roasted instant coffee	No data	I-1	250	3.3	Roasted instant coffee (97.1%), green coffee (2.9%)	Poland
Cappuccino instant coffee	No data	I-2	130	13.0	Roasted instant coffee (9.5%)	Poland
Cappuccino instant coffee	No data	J-1	110	12.7	Roasted instant coffee (12%)	Poland
Cappuccino instant coffee	No data	J-2	110	13.0	Roasted instant coffee (7%)	Poland
Cappuccino instant coffee	No data	A-2	500	13.3	Roasted instant coffee (3%)	Holland
Cappuccino instant coffee	No data	A-3	500	15.0	Roasted instant coffee (3%)	Holland
Cappuccino instant coffee	No data	A-4	18	18.0	Roasted instant coffee (3%)	Holland
Chicory instant drink	–	K-1	100	6.0	Bio, roasted chicory root	Poland
Chicory instant drink	–	L	100	6.0	Bio, roasted chicory root	Poland
Chicory instant drink	–	M	100	6.1	Roasted chicory root	Poland
Chicory instant drink	–	J-3	200	6.3	Bio, roasted chicory root	Poland
Chicory instant drink	–	N-1	25	25.0	Roasted chicory root	Poland
Chicory instant drink	–	O	100	6.0	Bio, roasted chicory root	Belgium
Blend of chicory and coffee instant drink	No data	N-2	100	6.3	Chicory 60%, coffee 40%	Poland
Blend of chicory and coffee instant drink	No data	J-3	180	5.2	Chicory 30.8%, coffee 34.3%, oligofructose 33%	Poland
Blend of chicory and coffee instant drink	No data	P	100	4.0	Chicory 60%, coffee 40%	Poland
Blend of chicory and coffee instant drink	No data	Q	100	4.0	Chicory 60%, coffee 40%	Poland
Blend of chicory and coffee instant drink	No data	R	150	4.0	Chicory 60%, coffee 20%, barley 20%	Poland
Instant cereal coffee drink	–	S	200	4.0	Chicory 60%, coffee 38%	Germany
Instant cereal coffee drink	–	K-2	200	6.0	Rye and barley 72%, chicory, sugar beet	Poland
Instant cereal coffee drink	–	K-3	200	6.4	Roasted barley 50%, chicory 30%, spicle wheat 20%	Poland
Instant cereal coffee drink	–	T-1	4.2	4.2	Rye 60%, barley 20%, chicory and white beet 20%	Poland
Instant cereal coffee drink	–	T-2	4.2	4.2	Rye 50%, chicory 50%	Poland
Instant cereal coffee drink	–	U	12	12.0	Roasted barley and rye 16.5%, instant coffee 12%	Poland
Instant cereal coffee drink	–	W	120	7.0	Roasted barley	Italy
2-in-1 instant coffee	No data	C-6	8.0	8.0	Instant coffee 19%	Poland
2-in-1 instant coffee	No data	A-5	14	14.0	Instant coffee 9.9%	Germany
2-in-1 instant coffee	No data	X	18	18.0	Instant coffee 10%	Poland
3-in-1 instant coffee	No data	B-5	20	20.0	Instant coffee 10.1%	Poland
3-in-1 instant coffee	No data	Y	18	18.0	Instant coffee 11%	Poland
3-in-1 instant coffee	No data	Z	15	15.0	Instant coffee 15%	Poland
3-in-1 instant coffee	No data	A-6	16	16.0	Instant coffee 15%	Germany
3-in-1 instant coffee	No data	C-7	17	17.0	Instant coffee 12.2%	Poland

##### Estimation of Safety

1. Tolerable weekly intake (TWI) % and BMDL % were calculated according to the formulas [18]:
  $$\mathrm{\%TWI}=\frac{{\mathrm{EWI}}_{\mathrm{Cd}}\times 100}{\mathrm{TWI}}$$
  TWI value: 2.5 μg Cd kg^−1^ body weight per week [21]
  $$\mathrm{\%BMDL}=\frac{{\mathrm{EWI}}_{\mathrm{Pb}}\times 100}{\mathrm{BMDL}}$$
  BMDL values: BMDL01 was 10.5 μg Pb per kg^−1^ body weight per week and BMDL10 was 4.4 μg Pb kg^−1^ body weight per week [22]. The mean body weight was assumed as 70 kg.
  EWI (estimated weekly intake) was calculated according to the formula [18]:
  $$\mathrm{EWI}=\frac{\mathrm{mean}\;\mathrm{weekly}\;\mathrm{consumption}\times{\mathrm{Cd}\;\mathrm{or}\;\mathrm{Pb}\;\mathrm{content}}}{\mathrm{body\,weight}}$$
2. Chronic daily intake (CDI) of Cd or Pb was calculated according to the formula [23, 24]:
  $$\mathrm{CDI}=\frac{\mathrm{EDI}\times\mathrm{EFr}\times{\mathrm{ED}}_{\mathrm{tot}}}{\mathrm{body}\;\mathrm{weight}\times\mathrm{AT}}$$
  where EDI is the estimated daily intake of Cd and Pb, calculated on the basis of the mean weekly consumption of drinks (one, two or three cups) and mean level of Cd and Pb; EFr is the days of exposure frequency (365 per year); ED_tot_ is the exposure duration (56 years); and AT is the period of exposure (365 per year).
3. Target hazard quotient (THQ) was calculated according to the formula [23]:
  $$\mathrm{THQ}=\mathrm{CDI}/\mathrm{RfD}$$
  where CDI stands for daily intake of Cd or Pb with beverages.
  The reference dose (RfD) for Cd is 1 µg kg^−1^ of body weight per day, and for Pb, it is 3.5 µg kg^−1^ of body weight per day [25].
4. Hazard index (HI) was calculated from the formula [23]:
  $$\mathrm{HI}=\mathrm{THQCd}+\mathrm{THQPb}$$

### Statistical Analysis

Statistica 13.1 software was used for statistical analysis. The mean, minimum and maximum values, and the standard deviation (SD) were calculated and an analysis of variance was carried out. The calculations took into account three replications for each chemical analysis. Statistically significant differences (*P* < 0.05) were determined by one-way analysis of variance (ANOVA) using Duncan’s test.

## Results

### The Content of Cd and Pb in Instant Coffee, Instant Coffee Drinks and Instant Coffee Substitutes

The content of Cd and Pb in the analysed beverages is presented in Table 3. The highest (*P* < 0.05) content of Cd was measured in chicory coffee (3.202 ± 0.7 µg kg^−1^) and cereal coffee (3.072 ± 0.4 µg kg^−1^). It was also significant in the chicory and natural roasted coffee blend (2.820 ± 0.9 µg kg^−1^). The lowest (*P* < 0.05) content of Cd was found in cappuccino coffee drink and instant natural coffee less than 0.1 µg kg^−1^. The highest (*P* < 0.05) content of Pb was observed in instant natural coffee (82.6 ± 6.9 µg kg^−1^) and the lowest (*P* < 0.05) in the chicory and natural coffee blend (11 ± 1.9 µg kg^−1^).

**Table 3:** Content of Cd and Pb in instant coffee (*n* = 49)

	Instant coffee, *n* = 11	Green and roasted coffee, *n* = 6	Cappuccino, *n* = 6	Chicory instant drink, *n* = 6	Chicory and coffee, *n* = 5	Cereal coffee drink, *n* = 7	2-in-1 or 3-in-1, *n* = 8	ANOVA *P*
Cd, μg kg^−1^								
Mean	0.095^e^	0.549^c^	0.030^f^	3.202^a^	2.820^b^	3.072^ab^	0.195^d^	0.003
Maximum	0.137^e^	0.732^c^	0.070^f^	4.319^a^	4.012^a^	3.580^b^	0.351^d^	< 0.001
Minimum	0.067^f^	0.358^d^	< LOQ^g^	2.289^b^	1.987^c^	2.653^a^	0.124^e^	< 0.001
Median	0.085	0.585	0.040	3.024	2.471	2.840	0.151	
SD	0.027	0.156	0.030	0.736	0.940	0.409	0.095	
Variance analysis	0.001	0.024	0.001	0.542	0.883	0.167	0.009	
Pb, μg kg^−1^								
Mean	82.64^a^	70.94^b^	25.42^d^	41.85^c^	11.00^f^	41.00^c^	12.52^e^	0.002
Maximum	88.81^a^	85.12^a^	27.54^c^	48.91^b^	13.33^d^	44.69^b^	13.22^d^	< 0.001
Minimum	73.22^a^	54.32^b^	22.34^d^	35.82^c^	9.010^f^	38.78^c^	11.87^e^	0.001
Median	86.73	69.82	26.01	41.03	10.25	39.98	12.38	
SD	6.955	11.46	1.929	5.106	1.947	2.438	0.650	
Variance analysis	48.37	131.3	3.722	26.08	3.790	5.943	0.423	

Average values for 3 replications. Means with different superscripts in the same lines differ significantly at *P* < 0.05 by Duncan’s test. LOQ Cd = 0.004 mg kg^−1^; LOQ Pb = 0.03 mg kg^−1^

*SD*, standard deviation; *LOQ*, limit of quantitation

### The Safety of Drinking Instant Coffee, Coffee Drinks and Coffee Substitutes

**Fig. 1:**
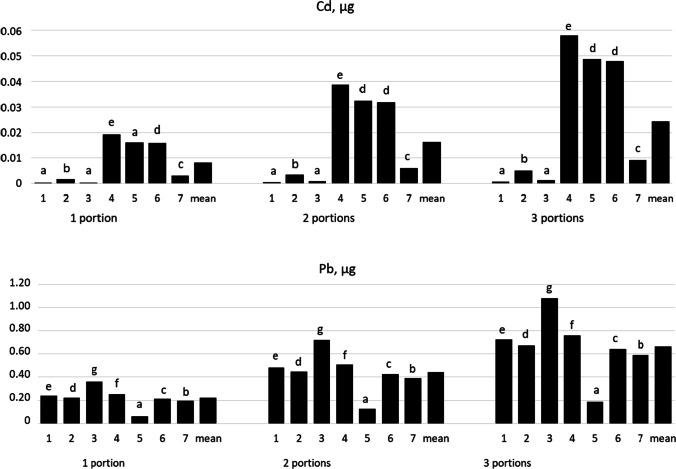
Mean Cd and Pb content in 1, 2 and 3 portions of instant coffee, coffee drinks and coffee substitutes. 1—instant coffee; 2—green and roasted coffee; 3—cappuccino; 4—chicory instant drink; 5—chicory and coffee; 6—cereal coffee drink; 7—2 in 1 and 3 in 1. Values with different superscripts differ at *P* < 0.05 by Duncan’s test

Table 4 presents data concerning the estimated safety of coffee drinks according to three consumption patterns (one, two or three servings of coffee a day). The content of Cd and Pb in coffee servings is presented in Fig. 1.

**Table 4:** Safety of coffee for consumption

	Instant coffee		Green and roasted coffee		Cappuccino		Chicory instant drink		Chicory and coffee		Cereal coffee drink		2 in 1 or 3 in 1	
	Cd	Pb	Cd	Pb	Cd	Pb	Cd	Pb	Cd	Pb	Cd	Pb	Cd	Pb
Pattern 1: drinking 1 cup of coffee a day														
EWI, µg^1^	0.002	1.685	0.012	1.564	0.003	2.521	0.135	1.767	0.114	0.443	0.112	1.492	0.021	1.369
% TWI^A,B^	0.001		0.007		0.002		0.077		0.065		0.064		0.012	
% BMDL01^A,D^		0.229		0.213		0.343		0.240		0.060		0.203		0.186
% BMDL10^A,C^		0.547		0.508		0.818		0.574		0.144		0.485		0.445
CDI^2^	3 × 10^−4^	0.254	0.002	0.236	4 × 10^−4^	0.381	0.020	0.267	0.017	0.067	0.017	0.225	0.003	0.207
THQ^3^	3 × 10^−4^	0.073	0.002	0.067	4 × 10^−4^	0.109	0.020	0.076	0.017	0.019	0.017	0.064	0.003	0.059
HI^4^	0.073		0.069		0.109		0.097		0.036		0.081		0.062	
Pattern 2: drinking 2 cups of coffee a day														
EWI, µg^1^	0.004	3.370	0.024	3.128	0.006	5.042	0.270	3.535	0.227	0.886	0.224	2.985	0.043	2.739
% TWI^A,B^	0.002		0.014		0.003		0.155		0.130		0.128		0.024	
% BMDL01^A,D^		0.463		0.430		0.693		0.486		0.122		0.410		0.376
% BMDL10^A,C^		1.094		1.016		1.637		1.148		0.288		0.969		0.889
CDI^2^	0.001	0.509	0.004	0.472	0.001	0.761	0.041	0.534	0.034	0.134	0.034	0.451	0.006	0.414
THQ^3^	0.001	0.145	0.004	0.135	0.001	0.218	0.041	0.153	0.034	0.038	0.034	0.129	0.006	0.118
HI^4^	0.146		0.139		0.218		0.193		0.072		0.163		0.125	
Pattern 3: drinking 3 cups of coffee a day														
EWI, µg^1^	0.006	5.054	0.036	4.693	0.009	7.562	0.406	5.302	0.341	1.328	0.335	4.477	0.064	4.108
% TWI^A,B^	0.003		0.021		0.005		0.232		0.195		0.192		0.036	
% BMDL01^A,D^		0.688		0.638		1.029		0.721		0.181		0.609		0.559
% BMDL10^A,C^		1.641		1.524		2.455		1.722		0.431		1.454		1.334
CDI^2^	0.001	0.763	0.005	0.709	0.001	1.142	0.061	0.801	0.051	0.201	0.051	0.676	0.010	0.620
THQ^3^	0.001	0.218	0.005	0.202	0.001	0.326	0.061	0.229	0.051	0.057	0.051	0.193	0.010	0.177
HI^4^	0.219		0.208		0.328		0.290		0.108		0.244		0.187	

^A^Mean body weight was assumed as 70 kg

^B^TWI—2.5 μg Cd per kg of body weight per week [21]

^C^BMDL01—10.5 µg Pb per kg of body weight per week [22]

^D^BMDL10—4.4 µg Pb per kg of body weight per week [22]

^1^EWI—estimated weekly intake calculated on the basis of the mean weekly consumption of coffee infusions and mean level of Cd and Pb

^2^Chronic daily intake calculated on the basis of the mean weekly consumption of coffee, mean level of Cd and Pb, and exposure duration

^3^Target hazard quotient calculated on the basis of the chronic daily intake of Cd or Pb

^4^Hazard index is the sum of THQ for Cd and Pb

#### Pattern 1: One Serving per Day

The EWI of Cd with instant coffee is 0.001 µg, which corresponds to 0.001% TWI; with cappuccino 0.003 µg (0.002% TWI); with a roasted coffee and green coffee blend 0.012 µg (0.007% TWI); with ‘2-in-1’ and ‘3-in-1’ blends 0.021 µg (0.012% TWI); with a chicory and natural coffee blend and cereal coffee about 0.11 µg (0.06% TWI); and with a chicory drink 0.135 µg, which corresponds to 0.077% TWI. In no case did CDI(Cd) and THQ(Cd) exceed 0.017. The highest estimated daily intake of Pb was observed for cappuccino, which corresponds to about 0.3% BMDL01 and about 0.8% BMDL10. For instant coffee, chicory drink and roasted and green coffee blend, EWI is ca. 1.6–1.7 µg (ca. 0.2% BMDL01 and ca. 0.5% BMDL10). Drinking cereal coffee will result in a Pb intake of 1.49 µg per week (ca. 0.2% BMDL01 and ca. 0.5% BMDL10), whereas EWI for a natural coffee and chicory blend is 0.44 µg (ca. 0.06% BMDL01 and 0.14% BMDL10). In no case did CDI(Pb) and THQ(Pb) exceed 0.4. The HI (Cd + Pb) ranged from 0.036 (chicory and coffee blend) to 0.109 (cappuccino).

#### Pattern 2: Two Servings per Day

The EWI of Cd with instant natural coffee amounted to 0.04 µg (0.002% TWI); with cappuccino 0.06 µg (0.03% TWI); with a roasted and green coffee blend 0.024 µg (0.014% TWI); and with ‘2-in-1’ and ‘3-in-1’ blends 0.043 µg (0.024% TWI). For other beverages, EWI was higher than 0.22 µg Cd (0.13–0.15% TWI). In no case did CDI(Cd) and THQ(Cd) exceed 0.05. For Pb consumed with a chicory and coffee blend, EWI was 0.886 µg (ca. 0.5% BMDL01 and ca. 1.1% BMDL10), with ‘2-in-1’ and ‘3-in-1’ blends ca. 3.7 µg (ca. 0.4% BMDL01 and ca. 0.9% BMDL10), and with other coffee drinks, EWI amounted to ca. 3.00–3.4 µg (ca. 0.4% BMDL01, ca. 1% BMDL10). Only with cappuccino was the EWI 5.042 µg, which corresponds to about 0.7% BMDL01 and more than 1.6% BMDL10. In no case did CDI(Pb) exceed 0.8 and THQ(Pb) 0.22. The HI (Cd + Pb) ranged from 0.072 (chicory and natural coffee blend) to 0.218 (cappuccino).

#### Pattern 3: Three Servings per Day

For Cd, EWI with instant coffee and with cappuccino is lower than 0.01 µg (max. 0.05% TWI), with a green and roasted coffee blend 0.036 µg (0.02% TWI), with ‘2-in-1’ and ‘3-in-1’ blends 0.064 µg (0.036% TWI), with a chicory and roasted coffee blend and with a roasted grain drink ca. 0.34 µg (ca. 0.2% TWI), and with a chicory drink more than 0.4 µg, which corresponds to above 0.2% TWI. In no case did CDI(Cd) and THQ(Cd) exceed 0.07. For Pb, EWI with a chicory and natural coffee blend amounted to ca. 1.3 µg (0.18% BMDL01, 0.4% BMDL10). With a green and roasted coffee blend and with a roasted grain drink and ‘2-in-1’ and ‘3-in-1’ blends, EWI was about 4.1–4.7 µg (max. 0.64% BMDL01, max. 1.5% BMDL10). With natural coffee and chicory drink, one can consume a little more than 5 µg Pb per week (ca. 0.7% BMDL01, max. 1.73% BMDL10), while with cappuccino more than 7.5 µg Pb (ca. 1.0% BMDL01, ca. 2.5% BMDL10). The value of CDI(Pb) did not exceed 0.8 and only for cappuccino was it above 1.15. The value of THQ(Pb) did not exceed 0.33. The HI (Cd + Pb) ranged from 0.108 (chicory and natural coffee blend) to 0.328 (cappuccino).

## Discussion

People like coffee drinks (instant coffee, cappuccino, coffee blends) and coffee substitutes (cereal coffee, decaffeinated coffee) since they can be easily prepared and taste good [1]. In Poland, instant coffee drinks are as popular as cereal coffee—being the choice of about 50% of coffee drinkers [1, 8, 12]. Nevertheless, it has not been thoroughly examined whether instant coffee is safe to drink, as implied by limited references on this subject. Analysis of available literature showed that only about 4% of all the references investigating the content of heavy metals in coffee refer to instant coffee. Perhaps this is due to the fact that instant coffee drinks are not popular in many countries; for instance, in the Balkans, the most popular choice is traditionally brewed coffee [26].

In our study, instant products containing natural coffee contained on average from six (a blend of green and roasted instant coffee) up to 100 (cappuccino) times less Cd per 1 kg than the beverages containing chicory and roasted grains. No directional relationship was identified for Pb between its content and the ingredients of the beverage; however, 100% coffee beverages (instant coffee, a blend of green and roasted coffee) contained several times more Pb than other analysed beverages did. The maximum level of Cd in the analysed beverages amounted to 4.3 µg (chicory instant drink), and that of Pb 88.8 µg (instant coffee) per 1 kg, which corresponds to 0.004 mg Cd and 0.089 mg Pb per 1 kg. Grembecka et al. [27] analysed as many as 120 samples of different kinds of coffee, including 27 samples of instant coffee. The abovementioned authors found that the content of Cd and Pb could not be determined using the applied method of analysis (LOD Cd = 0.003 mg 100 g^−1^, LOD Pb = 0.01 mg 100 g^−1^). Gogoasa et al. [28, 29] obtained similar results for several types of instant coffee available on the market in Romania. Studies by Alkherraz et al. [30] showed the content of Cd in instant coffee was ND–0.3 mg kg^−1^ and that of Pb was ND–3.9 mg kg^−1^. The maximum values were significantly higher than in our studies. Voica et al. [31] found instant coffee to contain a maximum of 0.002 mg Cd and 0.12–0.37 mg Pb. Studies into instant coffee drinks (2-in-1, 3-in-1, 4-in-1 and instant iced coffee) offered on the market in Serbia revealed that they contained a maximum of 0.01 ppm of Cd [26], so they are safe for consumers. In the abovementioned studies, determinable levels of Cd were measured only in 2 out of 15 samples of coffee drinks.

It should be emphasised that our studies found coffee drinks and coffee substitutes safe for consumers. Even with three servings a day of beverage containing the highest level of Cd (chicory drink, cereal coffee or a blend of chicory and natural coffee) or Pb (instant coffee, a blend of green and roasted coffee), the indicators of safety (CDI, THQ, HI) do not exceed 0.35 for Cd and 0.8 for Pb, which means that the risk of diseases related to chronic exposure to Cd and Pb consumed with coffee is very low. However, CDI(Pb) for cappuccino was higher than 1, which is a red flag; that was due to serving size, as one serving of cappuccino weighs more than 14 g, while for other beverages, it is about 3–4 times lower (except blends weighing more than 15 g). This poses a particular hazard as, during complex exposure (Cd + Pb), Pb accumulates in body organs at amounts higher than Cd, which was proved in studies involving rats [19]. It should be noted that cappuccino is one of the favourite coffee types in Poland, chosen by more than 84% of consumers [12]. In our studies, CDI(Pb) was also high for an instant chicory drink. Its value exceeded 0.8, and above 1 is considered a hazardous level. A chicory substitute of coffee is caffeine free and is suitable for children [32], which poses a particular risk to a young and sensitive body. Due to the presence of oligosaccharides (mainly inulin), a chicory drink has a pleasant, sweetish taste [33] that is accepted even by small children who show innate preferences for sweet taste [34].

In our studies, the percentage of tolerable intake of Cd and Pb with the analysed beverages did not exceed 2.5%, as recommended by EFSA [21, 22]. Other studies also corroborated that instant coffee drinks and their substitutes were safe for consumers [26, 27, 31]. It is worth noting that drinking instant coffee drinks and their substitutes is as safe as drinking roasted coffee brews, and numerous studies in many countries have corroborated that drinking roasted coffee is safe [17, 18, 35, 36]. However, it should be noted that some authors [17, 37] found Pb levels exceeding the tolerable value in as much as 75% of the analysed roasted coffee samples. Such information was not found in available literature for instant coffee. Our previous studies demonstrated that dry roasted coffee contained on average 3.78 μg Cd and 49.6 μg Pb per 1 kg [18]. In the abovementioned studies, on average, 95% of Cd and 94% of Pb penetrated into the solution, which means that one serving of coffee brew contained on average 0.16 µg Cd and 1.97 µg Pb, whereas one serving of instant coffee on average 0.008 µg Cd and 0.22 µg Pb (Fig. 1), that is, twenty times less Cd and nine times more Pb than in a ground coffee brew. Also, reviews by Pohl et al. [38] showed that dry roasted coffee contained several times more Cd and Pb than instant coffee did; considering the percentage of Cd and Pb penetrating into the beverage (94–95%), a roasted coffee brew contains more Cd and Pb than an instant coffee drink. For frequent coffee drinkers, this may have a significant effect on the intake of Cd and Pb with the diet, although it was demonstrated that drinking as many as three servings of roasted coffee per day (one serving: 6 g of ground coffee + 100 mL of water) still does not lead to exceeding the tolerable limits [18]. Differences in the coffee production process can give rise to differences in the content of heavy metals in instant and ground coffee. Ground coffee is made by roasting and drying coffee beans, while instant coffee is produced by evaporating water (through freeze-drying or spray drying) from a concentrated roasted coffee brew [39, 40]. Coffee brews usually contain smaller concentrations of elements than the corresponding roasted coffees from which the brews were prepared [38].

To sum up, the content of Cd and Pb in the analysed coffee beverages and coffee substitutes was low; thus, drinking such beverages is safe for consumers. However, no safe limits of heavy metal intake exist due to the ability of such metals to accumulate in living tissues (half-life up to 30 years), so beverages and food should be regularly monitored for heavy metals. With instant drinks, special note should be taken of Pb, as the level of this metal is higher than that of Cd, and for beverages with higher weight per serving (e.g. cappuccino), the intake of Pb can exceed consumer-safe levels if they are consumed on a regular basis. Therefore, it should be considered whether it is advisable that flavoured multi-ingredient instant coffee drinks be consumed from time to time only, and natural coffee with optional milk and/or sweeteners be the choice of regular coffee drinkers.
